# RIP3 is downregulated in human myeloid leukemia cells and modulates apoptosis and caspase-mediated p65/RelA cleavage

**DOI:** 10.1038/cddis.2014.347

**Published:** 2014-08-21

**Authors:** A-L Nugues, H El Bouazzati, D Hétuin, C Berthon, A Loyens, E Bertrand, N Jouy, T Idziorek, B Quesnel

**Affiliations:** 1 Inserm, U837, Institut pour la Recherche sur le Cancer de Lille, place de Verdun, Lille F-59045, France; 2 Service des Maladies du Sang, Centre Hospitalier et Universitaire de Lille, Rue Polonovski, Lille F-59045, France; 3 Université Lille Nord de France, Lille F-59045, France

## Abstract

The receptor-interacting protein kinase 3 (RIP3) associates with RIP1 in a necrosome complex that can induce necroptosis, apoptosis, or cell proliferation. We analyzed the expression of RIP1 and RIP3 in CD34+ leukemia cells from a cohort of patients with acute myeloid leukemia (AML) and CD34+ cells from healthy donors. RIP3 expression was significantly reduced in most AML samples, whereas the expression of RIP1 did not differ significantly. When re-expressed in the mouse DA1-3b leukemia cell line, RIP3 induced apoptosis and necroptosis in the presence of caspase inhibitors. Transfection of RIP3 in the WEHI-3b leukemia cell line or in the mouse embryonic fibroblasts also resulted in increased cell death. Surprisingly, re-expression of a RIP3 mutant with an inactive kinase domain (RIP3-kinase dead (RIP3-KD)) induced significantly more and earlier apoptosis than wild-type RIP3 (RIP3-WT), indicating that the RIP3 kinase domain is an essential regulator of apoptosis/necroptosis in leukemia cells. The induced *in vivo* expression of RIP3-KD but not RIP3-WT prolonged the survival of mice injected with leukemia cells. The expression of RIP3-KD induced p65/RelA nuclear factor-*κ*B (NF-*κ*B) subunit caspase-dependent cleavage, and a non-cleavable p65/RelA D361E mutant rescued these cells from apoptosis. p65/RelA cleavage appears to be at least partially mediated by caspase-6. These data indicate that RIP3 silencing in leukemia cells results in suppression of the complex regulation of the apoptosis/necroptosis switch and NF-*κ*B activity.

Impairment in cell death pathways represents a general characteristic of most cancer cells. Cells can die through several mechanisms; two such cell death pathways include apoptosis and necrosis, which display distinct characteristics.^1^ Necrosis can occur in either an incidental or intentional manner as a result of defined signals, and the term necroptosis has been proposed to describe this programmed necrosis.^2^ Activation of the receptor-interacting protein kinase 1 (RIP1) and 3 (RIP3) proteins in the necrosome complex can induce apoptosis, necroptosis, or cell proliferation after the activation of death receptors, including TNFR1, TRAIL, and FAS.^3, 4^ RIP1 and RIP3 are serine threonine kinases with strong homology.^5^ Both proteins are composed of a kinase domain at the N-terminus and a RIP homotypic interaction motif (RHIM) at the C-terminus of RIP3. The RIP1/RIP3 complex can induce necroptosis initiated by cell death receptors of the tumor necrosis factor family. RIP3 binds to RIP1 via their respective RHIM domains, and these proteins form a filamentous structure with characteristics similar to *β*-amyloids and can cross phosphorylate each other and several downstream targets involved in necroptosis, apoptosis, or nuclear factor-*κ*B (NF-*κ*B) activation.^6^

The role of RIP3 in necroptosis and inflammation has been extensively studied, but its role in cancer remains poorly understood. A previous study in chronic lymphocytic leukemia (CLL) showed that malignant lymphoid cells were resistant to tumor necrosis factor-*α* (TNF*α*+Z-VAD-induced (carbobenzoxy-valyl-alanyl-aspartyl-[*O*-methyl]-fluoromethylketone) necroptosis and expressed reduced levels of RIP3 and cylindromatosis (CYLD), which regulates RIP1.^7^ Another study on childhood acute lymphoblastic leukemia reported that RIP1 was necessary to mediate the inhibitor of apoptosis protein-mediated sensitization of blast cells to chemotherapy.^8^ Autocrine TNF*α* loops that activate NF-*κ*B through RIP1 have also been described in various cancer cell lines.^9, 10^

Here we report that the expression of RIP3 was decreased in the majority of acute myeloid leukemia (AML) patients examined, whereas the expression of RIP1 remained unaffected. The expression of a RIP3 mutant with an inactivated kinase domain (RIP3-kinase dead (RIP3-KD)) in myeloid cell lines resulted in massive and early apoptosis and the caspase-mediated cleavage of p65/RkelA at a caspase-6 putative consensus site. Moreover, only RIP3-KD prolonged the survival of leukemic mice. Our results show that RIP3 activity regulates the apoptosis/necroptosis switch via its kinase activity in leukemia cells, and that other functions of RIP3 that are independent of its kinase domain modulate apoptosis and NF-*κ*B activity.

## Results

### Reduced expression of RIP3 in acute myeloid leukemia blast cells

**Figure 1:**
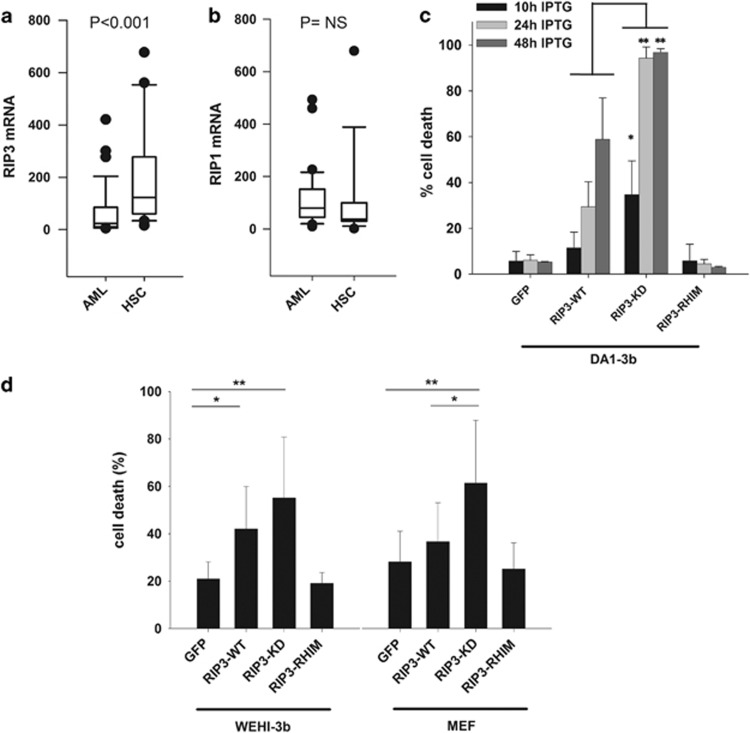
RIP3 is downregulated in AML and its expression in leukemia cell lines induced cell death. (**a**) Quantification of RIP3 mRNA via RQ-PCR in 32 sorted samples of CD34+ bone marrow blast cells from patients with AML compared with 26 samples of CD34+ hematopoietic cells from healthy donors. *P*<0.001 based on the Mann–Whitney rank sum test. (**b**) Same as **a** for RIP1. (**c**) Quantification of cell death via flow cytometry with propidium iodide (PI) in DA1-3b/GFP, DA1-3b/RIP3-WT, DA1-3b/RIP3-KD, and DA1-3b/RIP3-RHIM cells 10, 24, and 48 h after the addition of 1 mM IPTG. *P<1 × 10^−3^, **P<1 × 10^−4^, RIP3-KD *versus* RIP3-WT, based on the Mann–Whitney rank sum test. The graphs represent the mean±S.D. of 39 separate experiments at 10 h, 19 at 24 h, and 6 at 48 h. (**d**) Cell death in WEHI-3B leukemia cells and MEF measured as in **c**, 24 h after transfection with GFP, RIP3-WT, RIP3-KD, and RIP3-RHIM cDNA. The graphs represent the mean±S.D. of eight separate experiments. Student's *t*-test

To evaluate the expression of RIP1 and RIP3 in AML, we sorted CD34+ cells from a cohort of 31 patients with AML. AML CD34+ blast cells expressed significantly reduced RIP3 mRNA compared with CD34+ cells from healthy donors (Figure 1a), whereas the expression of RIP1 did not differ significantly (Figure 1b). The selection of blast cells via CD34+ sorting is mandatory, because several types of bone marrow cells (such as T lymphocytes) may naturally express high levels of these RIP proteins. This was confirmed through the observed higher RIP3 expression in the CD34-negative fractions of bone marrow cells from AML and healthy donors (Supplementary Figure S1). RIP3 expression did not correlate with any of the characteristics of the patients, but the size of the study cohort was not designed to explore differences between subgroups. Therefore, the necrosomes of AML blast cells revealed a selective defect in RIP3.

### The expression of RIP3-KD induces massive and early apoptosis

**Figure 2:**
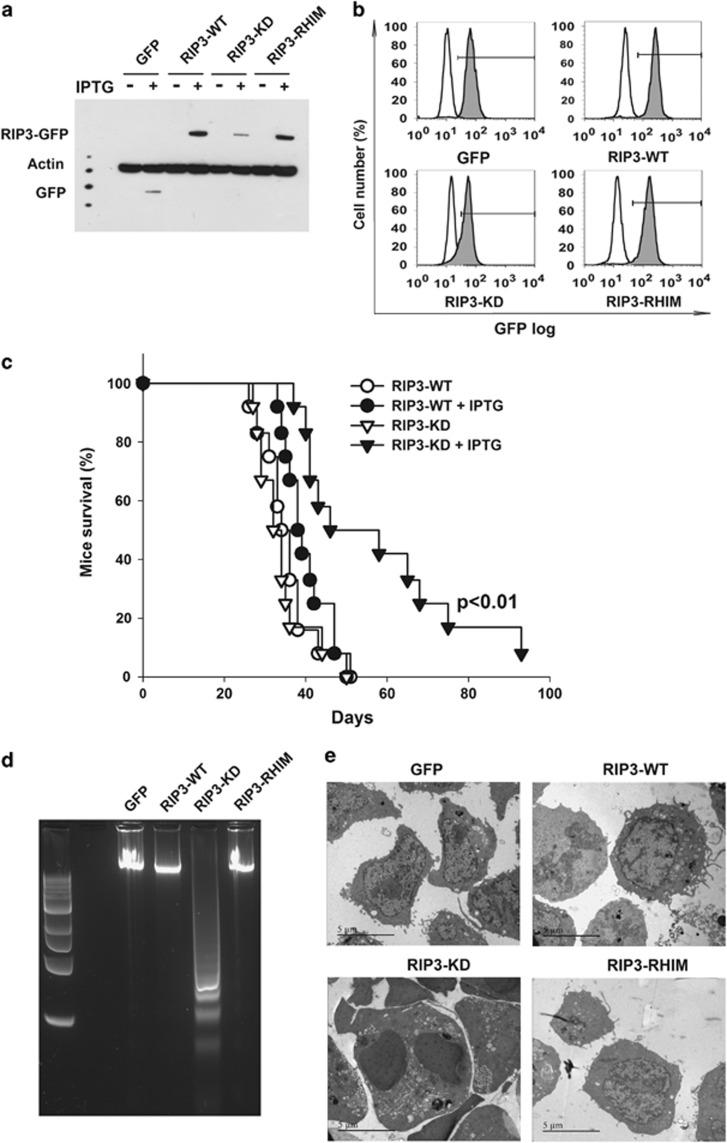
*In vitro* and *in vivo* expression of RIP3-WT and RIP3 mutants in DA1-3b cells. (**a**) Western blot analysis of GFP, RIP3-WT, RIP3-KD, RIP3-RHIM expression in DA1-3b cells 10 h after the addition of 1 mM IPTG. (**b**) The expression of RIP3-WT and RIP3 mutants in DA1-3b cells via flow cytometry 10 h after the addition of 1 mM IPTG. (**c**) Survival of mice injected intraperitoneally with 1 × 10^6^ DA1-3b/RIP3-WT and DA1-3b/RIP3-KD cells (10 mice/group). IPTG (12 mM) was added to the drinking water of the mice at day 10 (*P*<0.01, Log-rank test). (**d**) DNA fragmentation in DA1-3b/GFP, DA1-3b/RIP3-WT, DA1-3b/RIP3-KD, and DA1-3b/RIP3-RHIM cells 10 h after the addition of 1 mM IPTG. (**e**) Electron microscopy was performed on DA1-3b/GFP, DA1-3b/RIP3-WT, DA1-3b/RIP3-KD, and DA1-3b/RIP3-RHIM cells, which were analyzed 10 h after the addition of 1 mM IPTG. Representative images of live DA1-3b/GFP and DA1-3b/RIP3-RHIM cells and apoptotic DA1-3b/RIP3-WT and DA1-3b/RIP3-KD cells are shown. The images are shown at × 7000 magnification

To analyze the potential advantages for myeloid malignant cells due to reduced RIP3 expression, we induced the expression of wild-type RIP3 (RIP3-WT), RIP3-KD, and RIP3-RHIM mutants in the DA1-3b cell line. The RIP3-KD cDNA was generated with a mutation (D161N) resulting in the extinction of its kinase domain activity.^11^ The RIP3-RHIM cDNA was constructed with an AAAA-459-462 mutation to abolish the RIP3/RIP1 homotypic interaction.^11^ All of the cDNAs were fused to GFP to facilitate flow cytometry analysis.^11^ The DA1-3b cell line was generated through the transduction of BCR-ABL into a DA1 interleukin (IL)-3-dependent cell line.^12, 13^ DA1-3b cells are known to demonstrate high *in vivo* leukemogenicity and long-term persistence of minimal residual disease.^13, 14^ We observed that DA1-3b cells did not express RIP3 (Supplementary Figure S2). When RIP3 expression was induced by stably transfecting DA1-3b cells with the LacSwitch II Inducible Mammalian Expression System, cell death was observed 10 h after the addition of isopropyl *β*-D-1-thiogalactopyranoside (IPTG) and increased until 48 h, at which point the majority of the cell population showed a loss of viability (Figure 1c and Supplementary Figure S3). The induced expression of a RIP3-KD mutant with an inactivated kinase domain resulted in massive and more rapid cell death. The RIP3-RHIM mutant with an inactivating mutation in the homotypic interaction motif, which is necessary for the interaction with RIP1, showed no significant cell death, as observed for the GFP control (Figure 1c). Similar results were observed in the WEHI-3B mouse leukemia cell line and to a lesser degree in mouse embryonic fibroblasts (MEFs) (Figure 1d). The expression levels of the RIP3-WT and RIP3-RHIM proteins were similar after induction via IPTG. The RIP3-KD protein expression appeared to be lower, but the massive apoptosis observed after 10 h of IPTG made a strict comparison difficult (Figure 2a). Flow cytometry analysis confirmed that all RIP3 proteins were expressed with a slightly lower level for RIP3-KD, ruling out the possibility that the increased death rates observed for this mutant could result from enhanced expression levels (Figure 2b). An identical increase in cell death was observed in RIP3-KD-expressing cells in the parental DA1 cell line (devoid of the BCR-ABL construct) and in DA1-3b cells that were pre-incubated with a sublethal dose of imatinib, indicating that the mechanisms driven by RIP3-KD were not dependent on BCR-ABL-activated pathways (Supplementary Figure S4).

To strictly compare the effect of the *in vivo* expression of RIP3 and the different mutant proteins, we injected groups of C3H/HeOuJ mice with DA1-3b cells that had been transduced with RIP3-WT or RIP3-KD via an inducible system and added IPTG to the drinking water daily from day 10 until death. Only the induced expression of RIP3-KD significantly prolonged mouse survival (Figure 2c).

### RIP3-KD induces apoptosis

RIP3 is an essential mediator of cell death.^5^ When we analyzed DNA fragmentation in DA1-3b cells expressing RIP3-WT and the various mutants, it appeared that only cells expressing RIP3-KD showed typical DNA ladders 10 h after induction, suggesting that RIP3 lacking kinase activity induced apoptosis in DA1-3b cells (Figure 2d). An identical analysis 24 h after induction also showed DNA laddering in cells expressing RIP3-WT, but the bands were much more intense in the RIP3-KD cells (Supplementary Figure S5).

**Figure 3:**
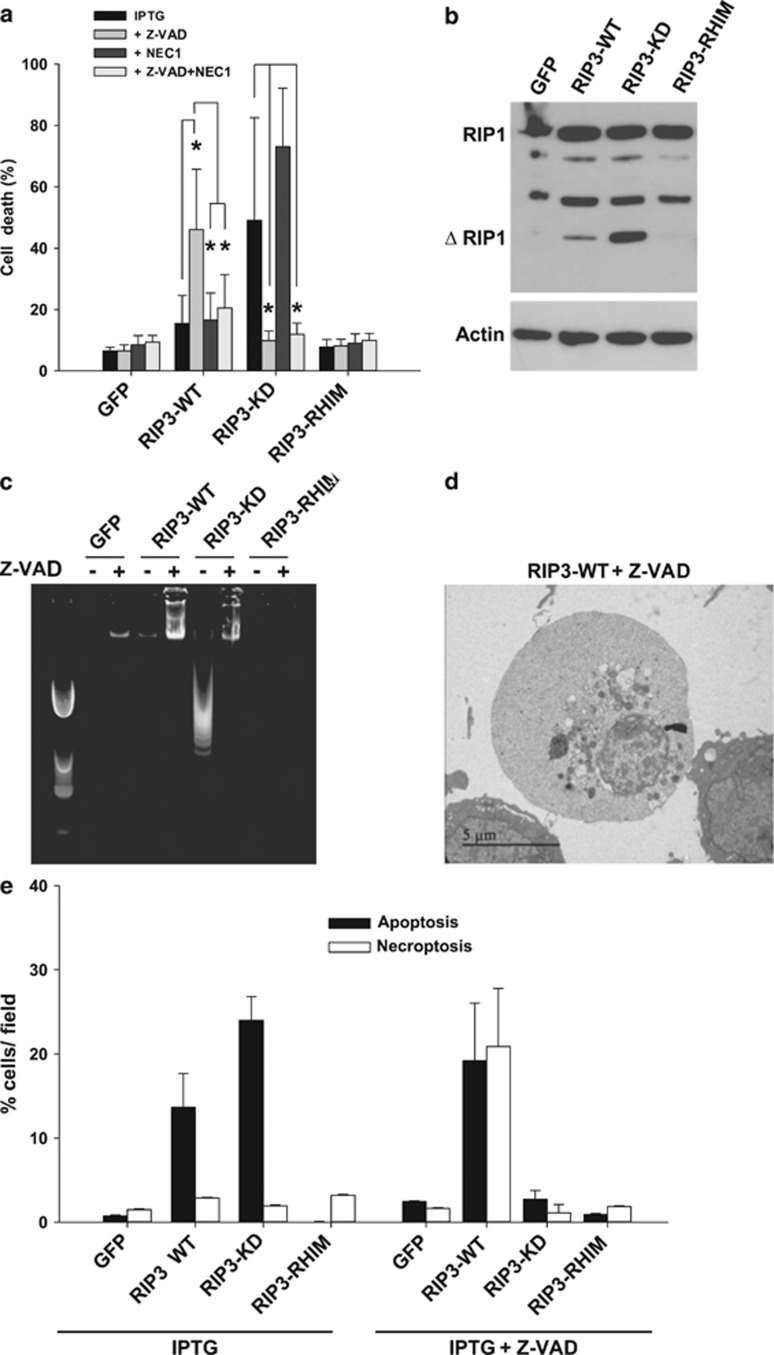
The necroptosis and apoptosis switch analysis in DA1-3b cells expressing RIP3-WT and RIP3 mutants. (**a**) Cell death quantification via flow cytometry in DA1-3b/GFP, DA1-3b/RIP3-WT, DA1-3b/RIP3-KD, and DA1-3b/RIP3-RHIM cells 10 h after the addition of 1 mM IPTG, 50 *μ*M Z-VAD, and 30 *μ*M NEC1. *P<1 × 10^−3^, based on the Mann–Whitney rank sum test. (**b**) Anti-RIP1 western blotting of DA1-3b/GFP, DA1-3b/RIP3-WT, DA1-3b/RIP3-KD, and DA1-3b/RIP3-RHIM cells 10 h after the addition of 1 mM IPTG. (**c**) DNA fragmentation assays performed 10 h after the addition of 1 mM IPTG +/−50 *μ*M Z-VAD to DA1-3b/GFP, DA1-3b/RIP3-WT, DA1-3b/RIP3-KD, and DA1-3b/RIP3-RHIM cells. (**d**) Electron microscopy was performed on DA1-3b/RIP3-WT cells 10 h after the addition of 1 mM IPTG+50 *μ*M Z-VAD. The images are shown at × 7000 magnification. (**e**) Quantification via electron microscopy of necroptosis and apoptosis in DA1-3b/GFP, DA1-3b/RIP3 WT, DA1-3b/RIP3-KD, and DA1-3b/RIP3-RHIM cells 10 h after the addition of 1 mM IPTG +/−50 *μ*M Z-VAD. Both characteristics of early and late apoptosis were counted as apoptotic cells. *P<1 × 10^−3^, based on the Mann–Whitney rank sum test. The graphs represent the mean±S.D. of three separate experiments performed in triplicate

To confirm that RIP3-WT and RIP3-KD induce apoptosis and not necroptosis, we examined DA1-3b cells via electron microscopy 10 h after the induction of either RIP3-WT or the RIP3 mutants using IPTG. The RIP3-KD cells showed clear signs of membrane blebbing and other typical characteristics of late apoptosis (Figures 2e and 3e). These indicators of apoptosis were also observed in RIP3-WT-expressing cells but were restricted to a smaller proportion of the cell population, and the indicators suggested less advanced stages of apoptosis (Figures 2e and 3e).

Activation of the necrosome complex in the presence of caspase inhibitors generally results in necroptosis.^3^ This phenomenon is hypothesized to be a back-up mechanism for cells that are infected by viruses capable of inactivating caspases. When DA1-3b cells were co-incubated with IPTG and the Z-VAD pan-caspase inhibitor, the RIP3-WT-expressing cells showed an increase in cell death with typical features of necroptosis (Figures 3a, d and e). The induction of RIP3-KD-mediated cell death was nearly totally suppressed by Z-VAD (Figure 3a). DNA fragmentation analyses confirmed these results, as less DNA laddering was observed in the RIP3-WT-expressing cells that were treated with Z-VAD (Figure 3c). Electron microscopy also showed that the RIP3-WT-expressing cells were both necroptotic and apoptotic in the presence of Z-VAD and that the apoptotic features of the RIP3-KD-expressing cells were abolished by Z-VAD (Figure 3e). Therefore, RIP3-KD induced caspase-dependent apoptosis that could not be converted to necroptosis via treatment with a caspase inhibitor.

We next determined whether RIP3-KD was dependent on RIP1. As previously reported, the expression of RIP3-WT induced RIP1 cleavage, and this cleavage was much more pronounced in the RIP3-KD cells (Figure 3b).^15^ The RIP1 kinase-specific inhibitor^2, 16^ necrostatin 1 (NEC1) abolished necroptotic RIP3-WT+Z-VAD-induced cell death (Figure 3a) and NEC1 had no effect on RIP3-KD-induced apoptosis. Together, these results indicate that RIP3 plays an important role in malignant myeloid cells that is independent of both its own kinase activity and the kinase activity of RIP1.

### NF-*κ*B antagonizes RIP3-KD-mediated cell death

**Figure 4:**
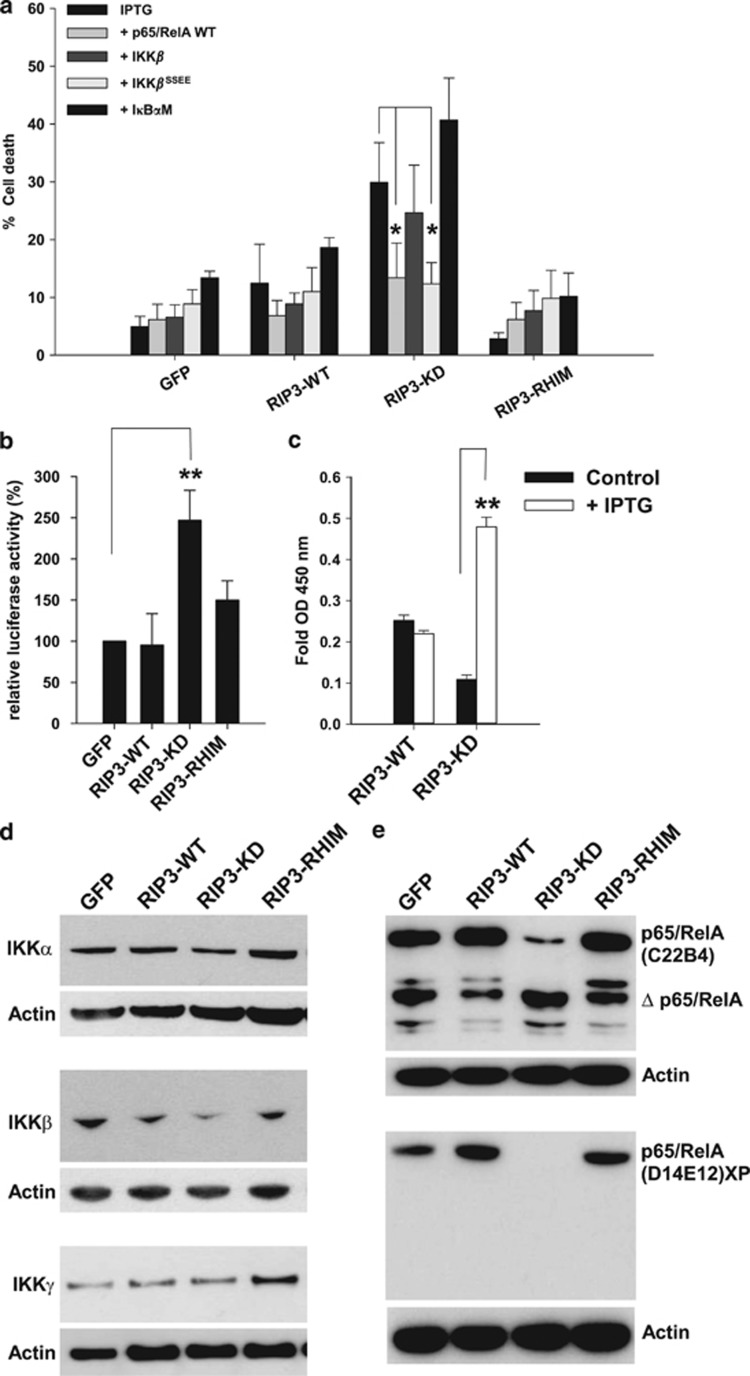
NF-*κ*B activity and p65/RelA cleavage. (**a**) Cell death quantification via flow cytometry 10 h after the addition of 1 mM IPTG to DA1-3b/GFP, DA1-3b/RIP3-WT, DA1-3b/RIP3-KD, and DA1-3b/RIP3-RHIM cells stably transfected with p65/RelA WT, IKK*β*, IKK*β*^SSEE^, or I*κ*B*α*M cDNA. *P<1 × 10^−3^, based on the Mann–Whitney rank sum test. The graphs represent the mean±S.D. of four separate experiments. (**b**) The transcriptional activity of NF-*κ*B was evaluated using a *κ*B-Luc reporter system 10 h after the addition of 1 mM IPTG, **P<1 × 10^−3^. (**c**) Quantification of NF-*κ*B complex DNA-binding activity in nuclear extracts from DA1-3b/RIP3-WT and DA1-3b/RIP3-KD cells 10 h after the addition of 1 mM IPTG, **P<1 × 10^−3^. (**d**) Western blot analysis of IKK*α*, IKK*β*, and IKK*γ* protein expression in DA1-3b/GFP, DA1-3b/RIP3-WT, DA1-3b/RIP3-KD, and DA1-3b/RIP3-RHIM cells 10 h after the addition of 1 mM IPTG. (**e**) Western blot analyses of p65/RelA expression using C22B4 or D14E12 XP antibodies in DA1-3b/GFP, DA1-3b/RIP3-WT, DA1-3b/RIP3-KD, and DA1-3b/RIP3-RHIM cells 10 h after the addition of 1 mM IPTG

RIP1 is involved in NF-*κ*B activation and the cell survival activated by TNFR1.^17, 18^ However, the role of RIP3 in NF-*κ*B activation remains controversial. Early reports suggested a role for NF-*κ*B, but further studies demonstrated that RIP3−/− cells presented normal NF-*κ*B activation through TNF*α* or Toll-like receptor stimulation.^19, 20, 21, 22^ Here we observed that the death of DA1-3b leukemia cells induced by RIP3-WT expression was not significantly affected in cells that were stably transfected with p65/RelA, I-kappa-B-kinase-beta (IKK*β*, or an IKK*β*^SSEE^ constitutively active mutant (Figure 4a). Moreover, a dominant-negative inhibitor kappa-B-alpha mutant (I*κ*B*α*M) demonstrated a modest additive effect on cell death. In sharp contrast, RIP3-KD-induced cell death was significantly antagonized by p65/RelA and IKK*β*^SSEE^. I*κ*B*α*M significantly increased cell death, but the marked individual effect of RIP3-KD did not enable us to distinguish between an additive or synergistic effect (Figure 4a). These results revealed that RIP3-KD-induced apoptosis but not RIP3-WT-induced apoptosis was dependent on NF-*κ*B activity.

To explore the hypothesis that RIP3-KD modulated NF-*κ*B activity, we analyzed the transcriptional activity of NF-*κ*B using a *κ*B-Luc reporter plasmid. The expression of RIP3 had no effect on NF-*κ*B activity, but the expression of RIP3-KD resulted in a significant increase in NF-*κ*B transcriptional activity 10 h after induction (Figure 4b). Analyses after 10 h were not relevant due to the nearly total cell death induced by RIP3-KD. These findings were confirmed by measuring the quantity of NF-*κ*B p65/RelA bound to consensus DNA-binding sites using a TransAM assay (Figure 4c).

### p65/RelA is cleaved in RIP3-KD-expressing cells

As NF-*κ*B activity appeared to antagonize RIP3-KD cell death, which contrasted with the apparent increase in NF-*κ*B activity observed after 10 h of RIP3-KD induction, we investigated whether the cascade of events initiated by RIP3-KD expression could affect specific components of NF-*κ*B. Although the expression of IKK*γ* (NEMO) was not affected by RIP3-KD expression, IKK*α* and IKK*β* appeared to be slightly reduced (Figure 4d). When we evaluated p65/RelA expression using the C22B4 mAb, we observed a specific decrease in the p65/RelA band in RIP3-KD-expressing cells (Figure 4e). A smaller band appeared to be markedly more prominent; this additional band was not observed when the D14E12XP mAb was used, but the decrease in p65/RelA protein was still observed in the RIP3-KD cells (Figure 4e).

**Figure 5:**
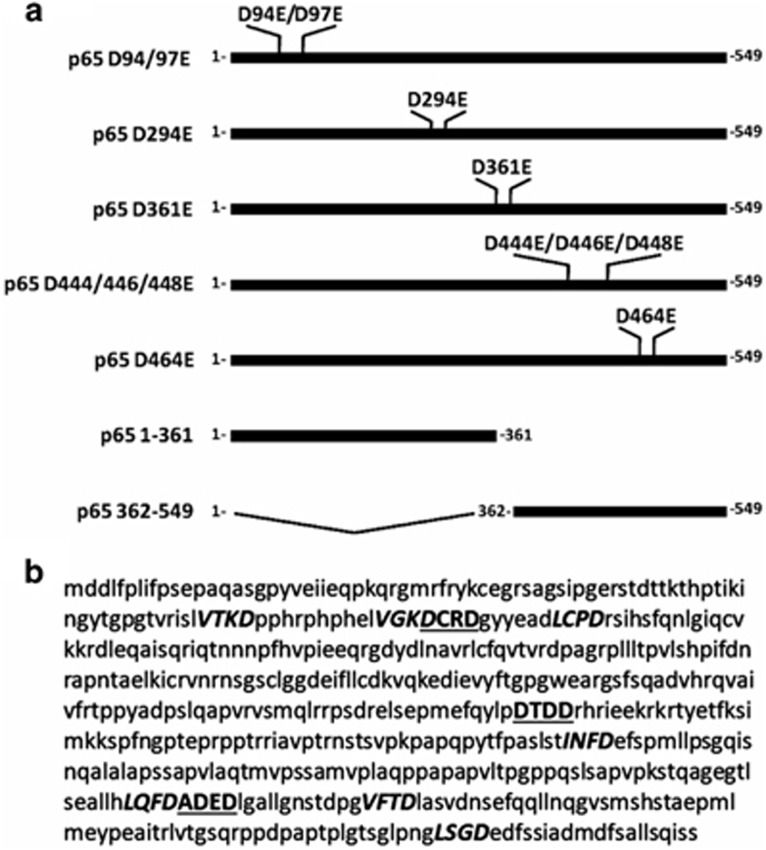
Construction of p65/RelA mutants at candidate caspase-dependent cleavage sites. (**a**) p65/RelA constructs with mutations in the candidate caspase-dependent cleavage sites and p65/RelA fragments 1–361 and 362–549 generated by p65/RelA cleavage at the D361 site. (**b**) Putative caspase-3 (bold underlined) and caspase-6 (bold italic) cleavage sites in the mouse p65/RelA protein

**Figure 6:**
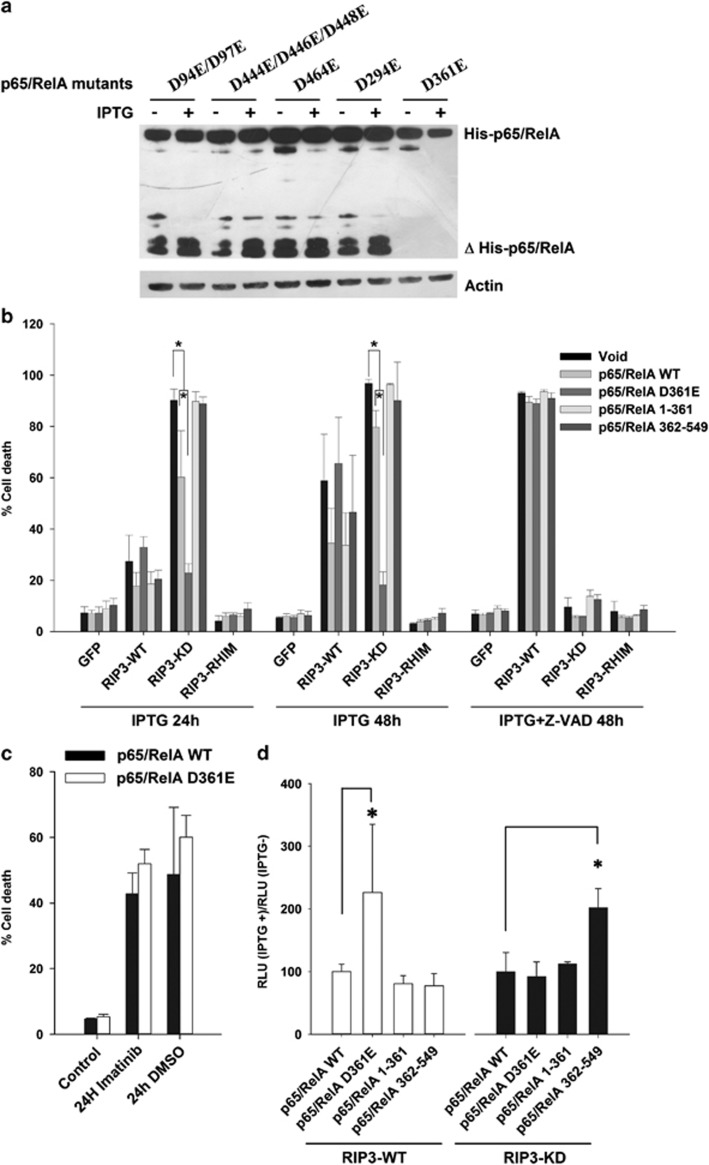
Resistance to cleavage and protection from RIP3-KD-mediated cell death by the p65/RelA D361E mutant. (**a**) Western blot analysis of p65/RelA expression with an anti-His-Tag antibody in DA1-3b/RIP3-KD cells that were transiently transfected with the p65/RelA mutants and incubated for 10 h with 1 mM IPTG. (**b**) Quantification of cell death 24 h (left panel) and 48 h (center panel) after the addition of 1 mM IPTG in DA1-3b/GFP, DA1-3b/RIP3-WT, DA1-3b/RIP3-KD, and DA1-3b/RIP3-RHIM cells that were stably transfected with p65/RelA WT, p65/RelA D361E, p65/RelA 1-361, or p65/RelA 362-549. The right panel is the same as the center panel but with the addition of 50 *μ*M Z-VAD. **P<1 × 10^−5^, IPTG *versus* p65/RelA, IPTG *versus* p65/RelA D361E, and p65/RelA *versus* p65/RelA D361E based on the Mann–Whitney rank sum test. (**c**) Cell death was measured in DA1-3b cells that were stably transfected with p65/RelA WT and p65/RelA D361E, and incubated with imatinib or DMSO for 24 h. The graphs represent the mean±S.D. of three separate experiments performed in triplicate. (**d**) Relative (IPTG+/IPTG−) transcriptional activity of NF-*κ*B evaluated using a *κ*B-Luc reporter system 10 h after the addition of 1 mM IPTG in DA1-3b/RIP3-WT (left panel) and DA1-3b/RIP3-KD (right panel) cells transfected with p65/RelA mutants as in **b**. *P<1 × 10^−3^. The graphs represent the mean±S.D. of three separate experiments performed in triplicate

This observed decrease in p65/RelA and the increased abundance of a smaller band in RIP3-KD cells suggested that p65/RelA may be cleaved. The caspase-dependent cleavage of p65/RelA has been previously described after the activation of apoptosis by TNF*α*, TRAIL, FAS, a chemical analog of naphthoquinone, HIV-1, or poliovirus infection.^23, 24, 25, 26^ These factors activate the proteolytic cleavage of p65/RelA by caspase-3 at consensus recognition sites. Seven putative recognition sites for caspase-6 (V/I/LXXD motif) and three putative caspase-3 sites (DXXD motif) have been reported in p65/RelA^27^ (Figure 5b). To confirm that RIP3-KD activated the caspase-dependent cleavage of p65/RelA and to analyze the specificity of the smaller band observed with the C22B4 mAb, we generated five p65/RelA constructs that were mutated in their caspase-3 or -6 consensus recognition sites and contained His-tags in their C-terminus (Figure 5a). The expression of RIP3-KD in DA1-3b cells that were transiently transfected with these mutants showed that only the p65/RelA D361E mutant was resistant to cleavage (Figure 6a). In addition, a smaller band, which was comparable in size with the previously observed band, was detected when the lysates were probed with an anti-His-tag mAb, confirming that p65/RelA was cleaved. Moreover, stable transfection of a p65/RelA D361E mutant abolished RIP3-KD cell death even after 48 h of induction of RIP3-KD (Figure 6b). Transfection of the p65/RelA 1–361 and 362–549 fragments resulting from the p65/RelA cleavage had no effect on the survival of the DA1-3b cells. Under conditions of necroptosis induced via RIP3-WT+Z-VAD, p65/RelA D361E had no effect (Figure 6b). When the expression of RIP3-WT was induced, the results mirrored those obtained with RIP3-KD (Figure 6b); the p65/RelA D361E mutant had no effect and the p65/RelA fragments led to slightly reduced cell death after 48 h (Figure 6b). The protective effect of the p65/RelA D361E mutant against apoptosis was specific to RIP3-KD-induced cell death because no change in cell death was observed when apoptosis was instead induced via treatment with imatinib or DMSO (Figure 6c).

p65/RelA fragments induced by caspase-3-mediated cleavage have been previously reported to have dominant-negative effects on the transcription of NF-*κ*B.^27^ When we analyzed the transcriptional effects of p65/RelA 1–361 and 362–549, no significant changes could be observed when compared with those of p65/RelA WT in RIP3-WT-expressing cells (Figure 6d). The p65/RelA D361E mutant showed significantly higher transcription than p65/RelA WT. The p65/RelA 362–549 fragments significantly increased NF-*κ*B transcription in the RIP3-KD cells; p65/RelA 1–361 showed no significant differences from p65/RelA WT. Thus, the p65/RelA fragments did not appear to directly inhibit NF-*κ*B transcription.

### p65/RelA cleavage by caspases

**Figure 7:**
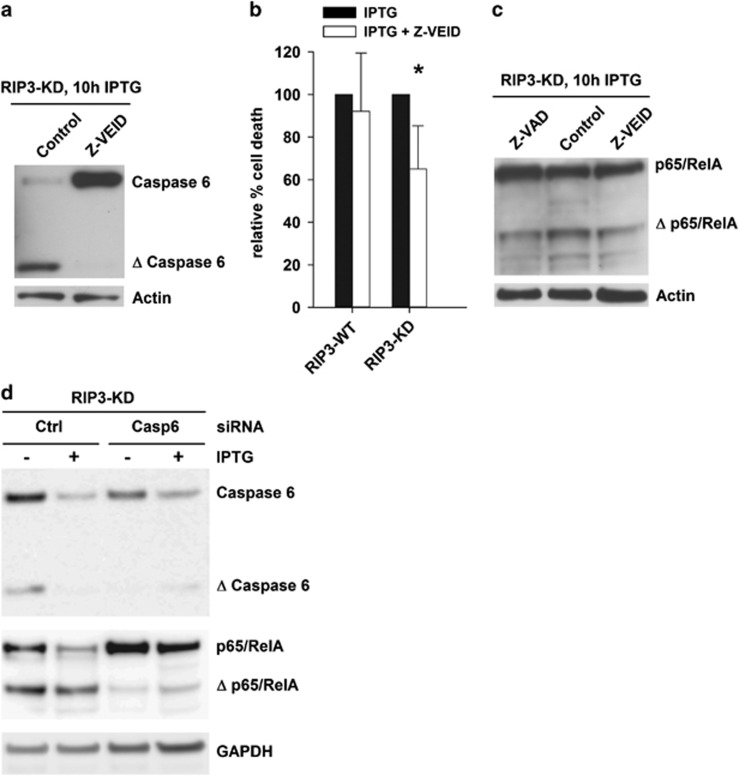
Caspase-6 activity in RIP3-KD-expressing cells. (**a**) Western blot analysis of caspase-6 expression and cleavage in DA1-3b/RIP3-KD cells 10 h after the addition of 1 mM IPTG. (**b**) The relative percentage of cell death measured via flow cytometry in DA1-3b/RIP3-WT and DA1-3b/RIP3-KD cells 10 h after the addition of 1 mM IPTG and 50 *μ*M Z-VEID (caspase-6 inhibitor). *P=0.04 Student's *t*-test. The graphs represent the mean±S.D. of three separate experiments (**c**) Western blot analysis of p65/RelA expression with C22B4 mAb 10 h after the addition of 1 mM IPTG and Z-VEID or Z-VAD. (**d**) p65/RelA and caspase 6 expression in DA1-3b cells 10 h after the addition of 1 mM IPTG and 24 h after transfection with caspase 6 (Casp6) or control (Ctrl) siRNAs

The p65/RelA D361E mutant was generated by mutating the *INFD* putative consensus recognition site for caspase-6. Western blot analysis showed that caspase-6 was expressed in DA1-3b cells and cleaved in RIP3-KD-expressing cells (Figure 7a). The caspase-6 inhibitor benzyloxycarbonyl-Val-Glu(OMe)-Ile-Asp(OMe)-fluoromethylketone (Z-VEID) partially reduced the cell death induced by RIP3-KD and slightly reduced p65/RelA cleavage (Figures 7a–c); the pan-caspase inhibitor Z-VAD had the same effect. Caspase 6 siRNA also reduced p65/RelA cleavage (Figure 7d). Therefore, the p65/RelA cleavage induced by RIP3-KD expression may have been mediated by caspase-6, but it appears likely that other proteases were also involved.

## Discussion

The role of necrosome complex components in cancer has only recently been explored.^5^ We observed here that CD34+ blast cells from patients with AML expressed significantly less RIP3 than CD34+ hematopoietic cells from healthy donors. This decreased expression was only observed in CD34+ selected blast cells and not in the whole bone marrow mononuclear cell fraction. Many normal cells naturally express RIP3, and the varied blast infiltration of the bone marrow in AML is likely to explain why the decreased expression of RIP3 may have been missed in previous studies. The alteration of the necrosome complex in hematological malignancies has only been reported in CLL, where the decreased expression of RIP3 and CYLD leading to a decreased sensitivity to TNF*α* were observed; however, only the role of CYLD in this process was specifically explored.^7^

Expression of RIP3 proteins in leukemia cell lines and MEFs showed that RIP3-WT induced cell death with much more pronounced efficiency of the KD mutant RIP3-KD. As previously reported by several studies, a mutation in the RIP3 RHIM domain abolished cell death induction.^3^

The RIP3-KD mutant induced massive and rapid apoptosis that was independent of RIP1 kinase activity in DA1-3b mouse leukemia cells (in which RIP3 is naturally silenced). RIP3-WT also induced apoptosis, but in a lower proportion of cells, and the induction of apoptosis was delayed compared with the apoptosis induced by RIP3-KD. Moreover, only RIP3-KD expression was able to prolong the survival of leukemic mice. Only the expression of RIP3-WT in the DA1-3b cells with inactivated caspases led to necroptosis. RIP3-KD did not induce necroptosis in the presence of a caspase inhibitor, indicating that the kinase domain of RIP3 plays an essential role in the apoptosis/necroptosis switch in malignant myeloid cells.

The surprisingly dramatic induction of apoptosis by RIP3-KD had not been previously observed in leukemia cells. Although the role of RIP3 in apoptosis remains unclear, the normal response of RIP3−/− thymocytes to apoptotic signals suggested that RIP3 does not play a role in this type of cell death.^22^ However, other studies have reported that the transduction of HeLa and 293T cell lines with RIP3 mutants that were either kinase-inactive or truncated by caspase-8 resulted in enhanced apoptosis.^28^ Newton *et al.*^29^ recently demonstrated that engineered mice expressing RIP3-KD (also with the D161N mutation) promoted lethal apoptosis. The RIP3−/− mice were viable, but the RIP3 KD/KD mice died at approximately embryonic day. Inducing the expression of RIP3-KD in adult mice resulted in massive apoptosis in the intestine and lymphocytes with intense cleaved caspase-3 staining. Moreover, they observed that the cell death induced by RIP3-KD was independent of CYLD and MLKL, which are essential mediators of necroptosis.^29, 30, 31, 32^ These data obtained *in vivo* in physiological tissues are similar to our results in leukemia cells. Thus, RIP3 is also a mediator of apoptosis.

The RIP1 kinase-specific inhibitor NEC1 abolished necroptotic RIP3-WT+Z-VAD-induced cell death, and NEC1 had no effect on RIP3-KD-induced apoptosis in the DA1-3b cells. These data are consistent with the known function of RIP1 kinase activity in the induction of necroptosis.^4^ Newton *et al.*^29^ demonstrated that a catalytically inactive RIP1 D138N mutant did not protect from apoptosis induced by RIP3-KD. The RIP1 kinase activity appears to be dispensable in RIP3-mediated apoptosis.

Among the different functions mediated by the necrosome, activation of the NF-*κ*B pathway promotes cell survival. This function is mediated by RIP1, although the role of RIP3 remains controversial. Experiments with RIP3−/− fibroblasts and macrophages have shown that NF-*κ*B is unaffected.^22^ Although these reports are certainly valid under physiological conditions, these findings need to be explored in cancer cells where NF-*κ*B is frequently enhanced and deregulated. In addition, truncated and mutated forms of RIP3 without active kinase domains have been shown to significantly enhance the transcriptional activity of NF-*κ*B in 293T cells.^28^ We observed here that RIP3-KD-mediated apoptosis was antagonized by the activation of the NF-*κ*B pathway. In sharp contrast, the transcriptional activity of NF-*κ*B appeared to be increased in DA1-3b/RIP3-KD cells, similar to the findings of Feng *et al.*^28^ in 293T cells. This enhanced binding to *κ*B-binding sites was limited to p65/RelA, as P50, P52, RELB, and CREL showed no enhanced binding. The enhanced activity of NF-*κ*B preceding apoptosis has been previously reported, notably during viral infections,^24^ chemotherapy,^33^ or cytokine deprivation.^27^ One possible regulatory mechanism for NF-*κ*B is the caspase-dependent cleavage of p65/RelA,^23, 24, 25, 26, 27^ and we observed that p65/RelA was cleaved at a putative caspase-6 consensus recognition site. Moreover, this cleavage was dramatically enhanced in RIP3-KD-expressing cells. Notably, only the p65 D361E mutant was resistant to cleavage and protected the RIP3-KD cells from apoptosis; this cleavage was abolished through the substitution of an aspartate to a glutamate at D361 within the *INFD* site. Conversely, p65/RelA WT but not p65/RelA D361E partially protected the RIP3-WT cells. To our knowledge, this is the first observation under experimental conditions of a cleavage at this site, although the precise role for this cleavage remains unclear. However, the cell death inhibition mediated by the p65/RelA D361E mutant suggests a role for cleavage in apoptosis. Dominant-negative effects of p65/RelA fragments on NF-*κ*B transcriptional activity have been observed.^26, 27^ In this study, the p65/RelA N-terminal 1–362 fragment showed no significant effect, but the C-terminal 362–549 fragment containing the two transactivation domains showed enhanced NF-*κ*B transcriptional activity only in RIP3-KD-expressing cells. However, both fragments had no effect on the survival of RIP3-KD cells. Therefore, the functions of RIP3 that are independent of its kinase domain activity appear to be extremely complex. It can be hypothesized that RIP3 kinase domain-independent functions may activate NF-*κ*B transcriptional activity and that p65/RelA cleavage modifies another unknown function of this protein directly via reduction of the available p65/RelA or indirectly via subtle modifications of transcription induced by these fragments. Moreover, cleavage at the *INFD* site spares the two transactivation domains of p65/RelA in the 362–549 fragment, and a recent report showed that p65/RelA contains a 21–186 fragment that specifically modulates ribosomal protein S3-dependent NF-*κ*B transcription.^34^ Another plausible hypothesis is that the RIP3 kinase activity inhibits p65/RelA cleavage and maintains a subtle equilibrium.

The RIP3-KD-mediated cleavage of p65/RelA was at least partially mediated by caspases, notably caspase-6. This caspase has been shown to be involved in neural cells, but its role in malignant hematopoietic cells has not been extensively explored. It has been shown that nucleophosmin mutants specifically inhibit the activities of caspase-6 and -8, and notably reduce their differentiation activities in myeloid cells.^35^ Caspase-6 is also an upstream activator of procaspase-8, which can inhibit necroptosis by cleaving RIP1, RIP3, and CYLD once it is activated.^36^ Interestingly, the caspase-8-mediated cleavage of RIP3 has been shown to generate a truncated form of RIP3 that lacks kinase activity but enhances NF-*κ*B activation and caspase-dependent apoptosis.^28^ Here we showed that RIP3-KD induced apoptosis and cleaved p65/RelA at a caspase-6 consensus site, suggesting that caspase-6 may also act downstream of RIP3. Knockdown of caspase-6 reduced p65/RelA cleavage. However, the partial effects observed with caspase inhibitors strongly suggest that other proteases are also involved.

The results presented here show that a decrease in RIP3 expression in blast cells from AML may enable malignant cells to suppress several functions, including necroptosis, apoptosis, and the modulation of the NF-*κ*B pathway through the caspase-mediated cleavage of p65/RelA. In addition, our findings indicate that some of these functions are independent of the activity of the RIP3 kinase domain. However, further investigations are needed to dissect the intrinsic benefit of this mechanism in leukemia cell survival and to identify possible therapeutic targets.

## Materials and Methods

### Selection of the CD34+fraction from patients and RIP1/RIP3 RQ-PCR

Bone marrow mononuclear cells from 32 patients with AML were isolated via Ficoll–Hypaque centrifugation after the donors had provided informed consent in accordance with the Declaration of Helsinki. The characteristics of the patients are listed in Table 1. Bone marrow cells from healthy donors were collected during bone marrow aspiration for allogeneic stem cell transplantation. This study was approved by the IRB Tumorotheque du Centre Hospitalier et Universitaire de Lille, Hopital Calmette, Lille, France. The CD34-positive cell population was isolated using a human CD34 magnetic MicroBead Kit (Miltenyi Biotec, Auburn, CA, USA) (>95% purity) according to the manufacturer's instructions. RNA and retrotranscripts were produced using conventional methods. RIP1 and RIP3 real-time quantitative PCR (RQ-PCR) was carried out using TaqMan technology (Life Technologies, Saint Aubin, France) according to the manufacturer's instructions, and GAPDH was used as a reference gene. The relative expression of RIP3 and RIP1 was quantified using the ΔΔCT method.

### Reagents and antibodies

IPTG (Sigma-Aldrich, Saint Louis, MO, USA) was used at a final concentration of 1 mM. Z-VAD (50 *μ*M) was purchased from Bachem (Bubendorf, Switzerland), NEC1 (30 *μ*M) was obtained from Alexis (Enzo Life Science, Villeurbanne, France), and imatinib (500 nM) was purchased from Cayman Chemicals (Ann Arbor, MI, USA). The caspase-6 inhibitor I Z-VEID (50 *μ*M) was purchased from Calbiochem (Darmstadt, Germany). Caspase-6, NF-*κ*B p65/RelA (C22B4), NF-*κ*B p65/RelA (D14E12) XP, and RIP1 XP primary antibodies and the ECL anti-mouse and -rabbit IgG HRP-linked whole secondary antibodies were purchased from Cell Signaling Technologies (Beverly, MA, USA). The RIP3 antibody was purchased from Santa Cruz (Dallas, TX,USA), the GFP mAb was obtained from Roche Applied Science (Meylan, France), and the His-Tag mAb was obtained from Novagen (Madison, WI, USA). All of the primary antibodies were used at 1 : 1000 final dilutions, and the secondary antibodies were used at 1 : 5000 dilutions. Caspase 6 was inactivated via transfection of 200 nM/5 million cells with Flexitube caspase 6 or control siRNA (target sequence: 5′-AAGCTGCATTTCTGTCCCAAA-3′) (Qiagen, Courtaboeuf, France).

### Cell lines

The leukemic murine DA1-3b p210^BCR-ABL^ cell line and the DA1-3b/C3HeOuJ mouse model have been described previously.^12, 13, 37^ The parental DA1 cells (obtained from and established by Ihle (1985)) were maintained with 4 ng/ml mouse IL-3 (PeproTech, London, UK).

### Plasmids and p65/RelA mutants

The GFP, RIP3-WT, RIP3-KD, and RIP3-RHIM mutant cDNAs were kindly provided by Pr. Francis Ka-Ming Chan. The RIP3-KD cDNA was generated with a mutation (D161N) resulting in the extinction of its kinase domain activity.^11^ RIP3-RHIM cDNA was constructed with an AAAA-459-462 mutation to abolish the RIP3/RIP1 homotypic interaction.^11^ All of the cDNAs were fused to GFP to facilitate flow cytometry analysis.^11^ The mouse RIP3 cDNAs were cloned into the LacSwitch II Inducible Mammalian Expression System (Agilent Technologies, Santa Clara, CA, USA). Stably inducible DA1-3b cells were obtained after transfection using Amaxa technology (Lonza, Basel, Switzerland). The resulting inducible cells were designated as DA1-3b/GFP, DA1-3b/RIP3-WT, DA1-3b/RIP3-KD, and DA1/RIP3-RHIM. Conditional expression of RIP3 protein was induced via the addition of 1 mM IPTG to the cell medium.

Mouse p65/RelA cDNA was purchased from Origene (Rockville, MD, USA) and then subcloned into the pVITRO *blasti* plasmid (Invivogen, Toulouse, France) and fused to a His-tag. All p65/RelA mutants were generated via directed mutagenesis using specific In-Fusion PCR cloning system (Clontech Laboratories Inc., Mountain View, CA, USA) primers (Supplementary Table 1) and were also inserted in the pVITRO *blasti* plasmid. The p65/RelA 1-361 fragment was generated with a His-tag in its 3′-end and the p65 362–549 fragment was generated with a Myc tag in its 5′-end. The IKK*β* WT and IKK*β*^SSEE^ constitutively active mutant cDNAs were kindly provided byAbu-Amer and colleagues.^38^ The I*κ*B*α* mutant (Addgene plasmid 12407) dominant-negative mutant cDNA was purchased from Addgene (Cambridge, MA, USA) and kindly provided by Verma and colleagues.^39^ After transfection, the stable cell lines were obtained via selection with blasticidin and the cells were sorted with an EPICS Altra flow cytometer (Beckman Coulter, Pasadena, CA, USA) according to DsRed fluorescence.

### Cell death, apoptosis, and necroptosis measurement

After the expression of inducible GFP, RIP3-WT, RIP3-KD, and RIP3-RHIM, cell death was analyzed via flow cytometry following extemporaneous incubation with 2 *μ*g/ml propidium iodide (Sigma-Aldrich) or according to forward-scatter and side-scatter analysis. DNA fragmentation was analyzed using the Quick Apoptotic DNA Ladder detection Kit (Invitrogen, Toulouse, France) according to the manufacturer's instructions.

The quantification of apoptotic and necroptotic cell death was carried out using electron microscopy.

### NF-*κ*B reporter assays

The *κ*B-luc reporter, which contains three *κ*B-binding sites, and negative control plasmids were kindly provided by Seuningen and colleagues.^40^ Five million inducible DA1-3b cells were transfected with the NF-*κ*B reporter vector or a negative control vector. Eight hours after transfection, the expression of RIP3 was induced with 1 mM IPTG for 10 h. A reporter assay was carried out according to previously published methods.^40^

The DNA-binding activities of NF-*κ*B p50, p52, p65/RelA, c-Rel, and RelB in nuclear extracts were detected using a TransAM NF-*κ*B family kit (Active Motif, Carlsbad, CA, USA) according to the manufacturer's instructions.

### *In vivo* experiments

Seven- to eight-week-old C3H/HeOuJ female mice (Charles River Laboratories, Lyon, France) were injected intraperitoneally with 1 × 10^6^ DA1-3b/RIP3-WT or DA1-3b/RIP3-KD-inducible cells. IPTG treatment was initiated 10 days after cell injection and consisted of the addition of 12 mM IPTG to the drinking water, which was made available to the mice daily until death. All animal experiments were approved by the Animal Care Ethical Committee CEEA.NPDC (Agreement no. AF-03-2008).

## Figures and Tables

**Table 1:** Patient characteristics

Total number of patients	32
Sex ratio	1.17
Median age (range)	59 (23–85)
	
*FAB*	
M0	3
M1	7
M2	12
M3	1
M4	1
M5	5
M6	1
AML evolved from MDS	2
	
*Karyotype:*	
Good	9
Intermediate	13
Poor-risk	10

## Abbreviations

ALL: acute lymphoblastic leukemia
AML: acute myeloid leukemia
Z-VEID: Benzyloxycarbonyl-Val-Glu(OMe)-Ile-Asp(OMe)-fluoromethylketone
Z-VAD: carbobenzoxy-valyl-alanyl-aspartyl-[O-methyl]-fluoromethylketone
CYLD: cylindromatosis
IKKb: I-kappa-B-kinase-beta
IKKg: I-kappa-B-kinase-gamma
IkBaM: inhibitor kappa B alpha mutant
IPTG: Isopropyl *β*-D-1-thiogalactopyranoside
MEF: mouse embryonic fibroblasts
NEC1: necrostatin 1
NF-κB: nuclear factor κ-B
RIP1, RIP3: receptor-interacting protein kinase 1 and 3
RHIM: RIP homotypic interaction motif
RIP3-KD: RIP3-kinase dead
TNF*α*: tumor necrosis factor alpha
NPM: nucleophosmin
PI: propidium iodide
SSC: side scatter
RQ-PCR: real-time quantitative PCR
