# Supplementary material for: RIP3 is downregulated in human myeloid leukemia cells and modulates apoptosis and caspase-mediated p65/RelA cleavage
Source: Cell Death Dis. 2014 Aug 21;5(8):e1384–. doi: 10.1038/cddis.2014.347 (PMC4454320; doi:10.1038/cddis.2014.347)
Supplement: Supplementary Table 1 [file cddis2014347x1.pdf]

Supplemental Table 1: Primers used to generate the p65/RelA mutants used in this study.

p65/RelA mutant	Primers
p65/RelA D94-97E	R1 3' CATAGTAGCCTTCCCGGCATTCCTTC 5'<br>F2 5' GAAGGAATGCCGGGAAGGCTACTATG 3'
p65/RelA D444-446-448E	R1 3' GCCCCCAACTCTTCTTCAGCTTCAAAC 5'<br>F2 5' GTTTGAAGCTGAAGAAGAGTTGGGGGC 3'
p65/RelA D464E	R1 3' CACAGATGCCAGTTCTGTGAACACTCC 5'<br>F2 5' GGAGTGTTCACAGAACTGGCATCTGTG 3'
p65/RelA D294E	R1 3' AATCCGGTGGCGTTCATCTGTGTCT 5'<br>F2 5' AGACACAGATGAACGCCACCGGATT 3'
p65/RelA D361E	R1 3' GGGGGAAAACTCTTCAAAGTTGATGG 5'<br>F2 5' CCATCAACTTTGAAGAGTTTTCCCCC 3'
