# Supplementary material for: RIP3 is downregulated in human myeloid leukemia cells and modulates apoptosis and caspase-mediated p65/RelA cleavage
Source: Cell Death Dis. 2014 Aug 21;5(8):e1384–. doi: 10.1038/cddis.2014.347 (PMC4454320; doi:10.1038/cddis.2014.347)
Supplement: Supplementary Figure S1 [file cddis2014347x2.pdf]

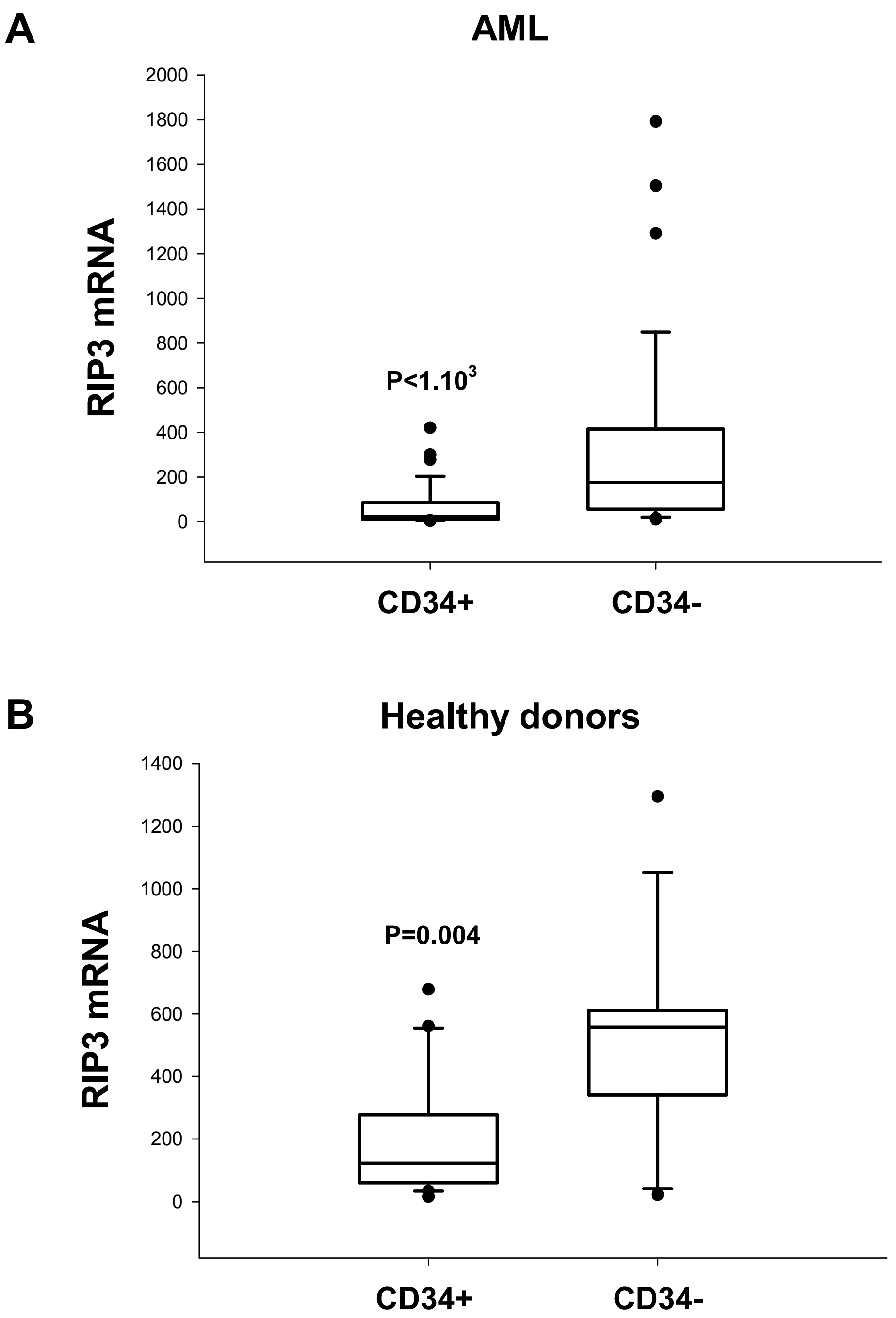

Supplementary Figure S1: RIP3 expression in CD34+ and CD34- bone marrow mononuclear cells. (A) Quantification of RIP3 mRNA by RQ-PCR in 32 sorted samples of CD34+ and CD34- bone marrow blast cells from patients with AML. (B) Same as (A) but in 26 samples of bone marrow mononuclear cells from healthy donors. Statistics based on Mann-Whitney Rank Sum Test.
