# Supplementary material for: RIP3 is downregulated in human myeloid leukemia cells and modulates apoptosis and caspase-mediated p65/RelA cleavage
Source: Cell Death Dis. 2014 Aug 21;5(8):e1384–. doi: 10.1038/cddis.2014.347 (PMC4454320; doi:10.1038/cddis.2014.347)
Supplement: Supplementary Figure S2 [file cddis2014347x3.pdf]

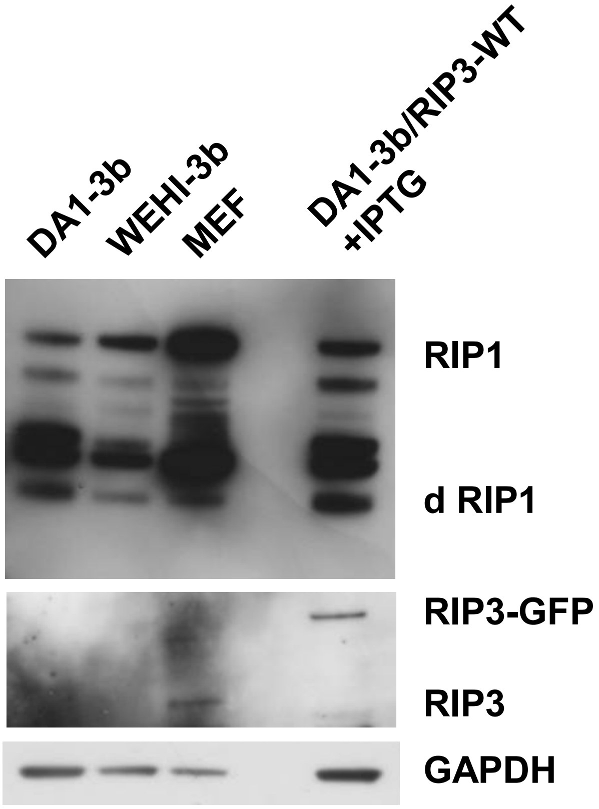

Supplementary Figure S2: Absence of RIP3 expression in DA1-3b and WEHI-3B cells. Anti-RIP1 and RIP3 western blot analyses of DA1-3b, WEHI-3b, MEF and DA1-3b/RIP3-WT + 1 mM IPTG for 10 h.
