# Supplementary material for: RIP3 is downregulated in human myeloid leukemia cells and modulates apoptosis and caspase-mediated p65/RelA cleavage
Source: Cell Death Dis. 2014 Aug 21;5(8):e1384–. doi: 10.1038/cddis.2014.347 (PMC4454320; doi:10.1038/cddis.2014.347)
Supplement: Supplementary Figure S3 [file cddis2014347x4.pdf]

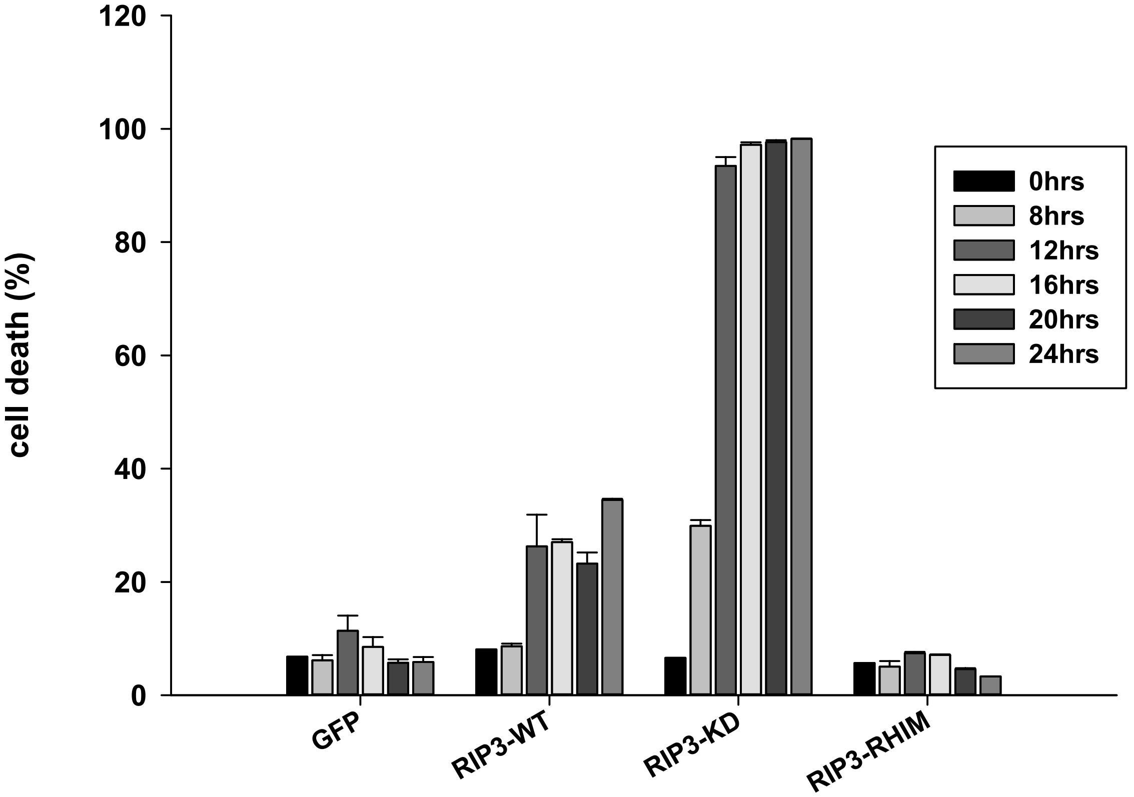

Supplementary Figure S3: Kinetic expression of RIP3 and mutant in DA1-3b cells. Quantification of cell death by flow cytometry with propidium iodide (PI) in DA1-3b/GFP, DA1-3b/RIP3-WT, DA1-3b/RIP3-KD, and DA1-3b/RIP3-RHIM cells after 8, 12, 16, 20, and 24 h after the addition of 1 mM IPTG. The graphs represent the mean ± s.d. of 3 separate experiments.
