# Supplementary material for: RIP3 is downregulated in human myeloid leukemia cells and modulates apoptosis and caspase-mediated p65/RelA cleavage
Source: Cell Death Dis. 2014 Aug 21;5(8):e1384–. doi: 10.1038/cddis.2014.347 (PMC4454320; doi:10.1038/cddis.2014.347)
Supplement: Supplementary Figure S4 [file cddis2014347x5.pdf]

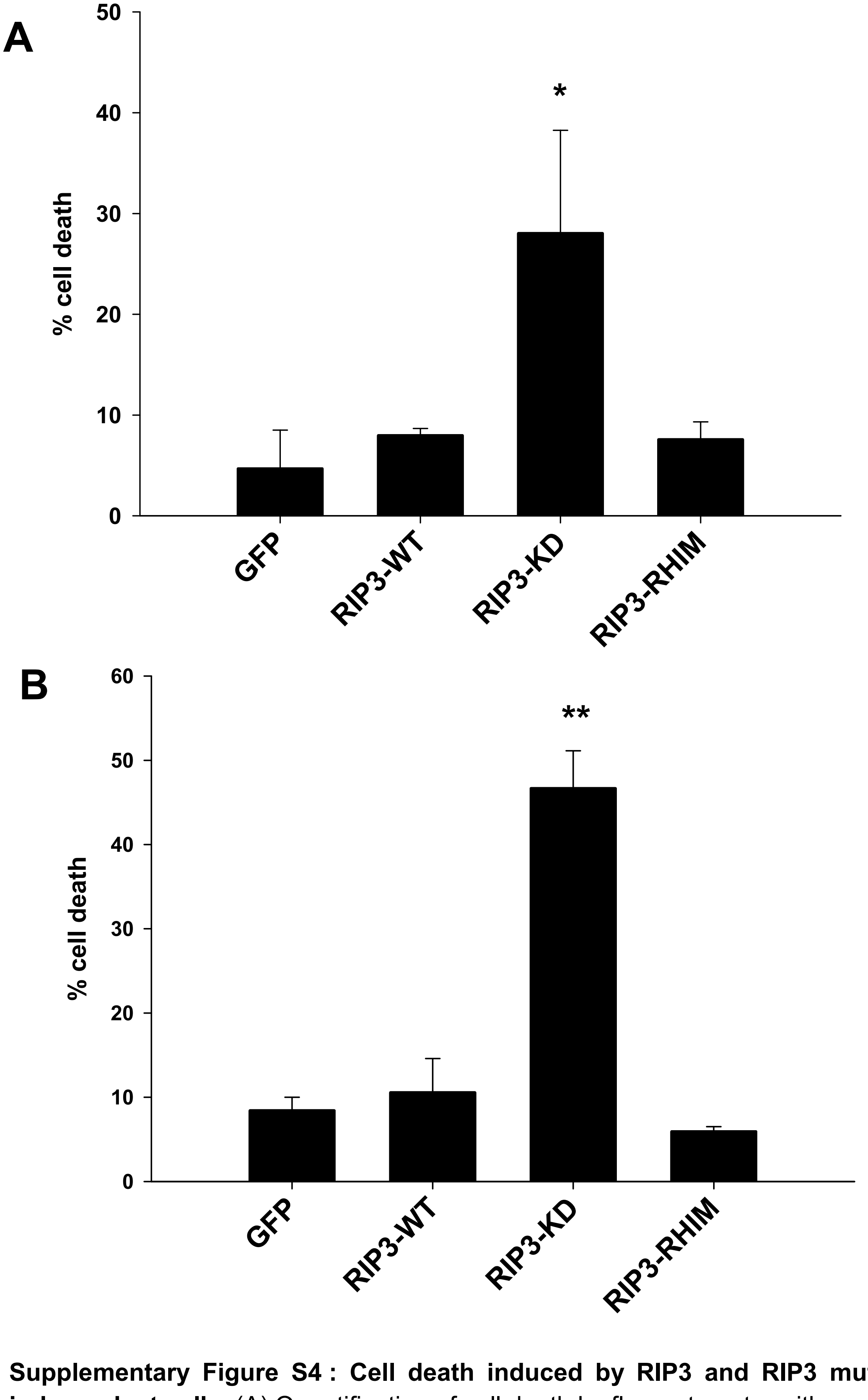

Supplementary Figure S4 : Cell death induced by RIP3 and RIP3 mutants in BCR-ABL independent cells. (A) Quantification of cell death by flow cytometry with propidium iodide (PI) in DA1/GFP, DA1/RIP3-WT, DA1/RIP3-KD , and DA1/RIP3-RHIM cells 10 h after the addition of 1 mM IPTG. (B) same as (A) but DA1-3b/GFP, DA1-3b/RIP3-WT, DA1-3b/RIP3-KD, and DA1-3b/RIP3-RHIM cells 10 h after the addition of 1 mM IPTG plus 100mM (sublethal dose) imatinib. * P=0.028; ** P< 1x 10^{-4}, based on Mann-Whitney Rank Sum Tast. The graphs represent the mean ± s.d. of 3 separate experiments.
