# Supplementary material for: RIP3 is downregulated in human myeloid leukemia cells and modulates apoptosis and caspase-mediated p65/RelA cleavage
Source: Cell Death Dis. 2014 Aug 21;5(8):e1384–. doi: 10.1038/cddis.2014.347 (PMC4454320; doi:10.1038/cddis.2014.347)
Supplement: Supplementary Figure S5 [file cddis2014347x6.pdf]

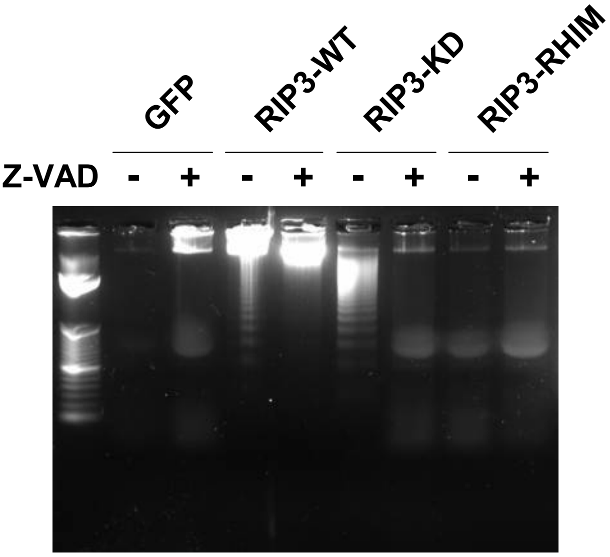

Supplementary Figure S5: RIP3-WT and RIP3-KD induce apoptosis in DA1-3b cells. DNA fragmentation assays performed 24 h after the addition of 1 mM IPTG to DA1-3b/GFP, DA1-3b/RIP3-WT, DA1-3b/RIP3-KD, and DA1-3b/RIP3-RHIM cells.
